# Activation of Melanocortin Receptors as a Potential Strategy to Reduce Local and Systemic Reactions Induced by Respiratory Viruses

**DOI:** 10.3389/fendo.2020.569241

**Published:** 2020-12-10

**Authors:** Caterina Lonati, Stefano Gatti, Anna Catania

**Affiliations:** Center for Preclinical Research, Fondazione IRCCS Ca’ Granda Ospedale Maggiore Policlinico, Milan, Italy

**Keywords:** respiratory viruses, SARS-CoV-2, acute lung injury, cytokine storm, endothelial dysfunction, melanocortin receptors, adenocorticotropin, alpha melanocyte stimulating hormone

## Abstract

The clinical hallmarks of infections caused by critical respiratory viruses consist of pneumonia, which can progress to acute lung injury (ALI), and systemic manifestations including hypercoagulopathy, vascular dysfunction, and endotheliitis. The disease outcome largely depends on the immune response produced by the host. The bio-molecular mechanisms underlying certain dire consequences of the infection partly arise from an aberrant production of inflammatory molecules, an event denoted as “cytokine storm”. Therefore, in addition to antiviral therapies, molecules able to prevent the injury caused by cytokine excess are under investigation. In this perspective, taking advantage of melanocortin peptides and their receptors, components of an endogenous modulatory system that exerts marked anti-inflammatory and immunomodulatory influences, could be an effective therapeutic strategy to control disease evolution. Exploiting the melanocortin system using natural or synthetic ligands can form a realistic basis to counteract certain deleterious effects of respiratory virus infections. The central and peripheral protective actions exerted following melanocortin receptor activation could allow dampening the harmful events that trigger the cytokine storm and endothelial dysfunction while sustaining the beneficial signals required to elicit repair mechanisms. The long standing evidence for melanocortin safety encourages this approach.

## Introduction

The present pandemic caused by Severe Acute Respiratory Syndrome (SARS)-Coronavirus (CoV)-2 follows outbreaks of other highly pathogenic respiratory viruses that emerged over the past two decades, namely SARS-CoV-1, Middle East respiratory syndrome (MERS)-CoV, and influenza A virus (IAV) H5N1, H1N1, and H7N9.

Clinical hallmarks of severe diseases are pneumonia with marked leukocyte infiltration into the lungs, diffuse alveolar damage (DAD), hyaline membrane formation, and edema (1–4). Pulmonary disease can progress to acute lung injury (ALI), acute respiratory distress syndrome (ARDS), and, in the most severe cases, to fibrosis (5). In addition, systemic manifestations are a major component of the clinical picture. Among these, endotheliitis with lymphocyte infiltration into vessel walls of the lung, heart, liver, and kidney (3, 6–8), deranged coagulation (2, 7, 9–11), and lymphopenia (4, 7, 12–14) significantly contribute to disease deterioration. There is also evidence for a viral spreading into the central nervous system (CNS) (7, 15, 16).

The bio-molecular mechanisms underlying clinical pathology partly arise from an aberrant release of inflammatory molecules, an event denoted as “cytokine storm”. Therefore, in addition to antiviral therapies, molecules able to prevent the consequences of the cytokine storm are under investigation (17–19). Use of corticosteroids was initially controversial due to the immunodepressive potential side effect (10, 14, 20–23), but their administration is now recommended in selected patients (24). In this perspective, taking advantage of melanocortin peptides and their receptors, components of an endogenous modulatory system, could be an effective and safe therapeutic strategy.

Melanocortins exert marked anti-inflammatory and immunomodulatory influences. Physiological role and effectiveness in treatment of acute, chronic, and systemic inflammatory disorders are well-documented (25–28). Exploiting the melanocortin system could form a realistic basis to counteract certain deleterious effects of respiratory viruses not only for the present but also for possible future pandemics.

## Pathological Consequences of Virus/Host Interactions

The outcome of CoVs and IAVs-induced disease largely depends on the immune response mounted by the host in response to infection (29–32). The term “cytokine storm” was firstly used to denote a hypercytokinemic state observed in H5N1-infected patients (33). Lung inflammation appears to be the primary “driver” (1–4) triggering excessive systemic production of interferons (IFN), interleukins (IL), chemokines, colony stimulating factors (CSF), and tumor necrosis factors (TNF) (19, 29, 30). There is now compelling evidence that the cytokine storm significantly contributes to both pulmonary and systemic damage associated with respiratory viral infections (19, 29, 30, 34, 35).

Cytokine storm arises from a virus-induced transient impairment of the physiological antiviral response. The inhibitory effects exerted by CoVs and IAVs on the host immunity are mediated by common mechanisms (14, 29, 36). Indeed, virus-encoded non-structural proteins are able to prevent immune recognition and IFN-dependent antiviral responses (19, 29, 31, 32, 37–39). Besides inhibitory effects, the virus also triggers host inflammatory signals (31, 39) through both activation of the nuclear factor (NF)-*κ*B and inflammasome formation (40, 41). The initial deficiency of the host immune response allows rapid viral replication and spreading in pulmonary cells. At later stages, deranged activation of the host immunity causes aberrant pulmonary secretion of IL-1ß, TNF-α, IL-6, IL-8, MCP-1, MIP-1*α*, Granulocyte macrophage (GM)-CSF, and Granulocyte (G)-CSF as well as massive recruitment of monocytes/macrophages and neutrophils into the lungs (14, 30–32, 36–38, 42).

When locally-produced inflammatory mediators reach the circulation, the clinical picture turns into systemic inflammation mainly due to activation of endothelial cells (43). Increased expression of adhesion molecules mediates leukocyte–endothelial adhesion leading to massive immune cell migration from the blood circulation into the extravascular connective tissue (34). Sustained inflammatory activation of endothelial cells can result in endothelial dysfunction (8) marked by exposure of tissue factor, aberrant release of Von Willebrand factor multimers, and decreased production of endogenous anticoagulants such as tissue factor pathway inhibitor (TFPI) and antithrombin (44). Hypercytokinemia likewise activates the hepatic acute phase response (34). In addition, increased blood concentration of CSFs stimulates the hematopoietic system to release both leukocytes and platelets in the circulation (34). These biological events further boost production of pro-inflammatory molecules contributing to amplify inflammation. At later stages, high TNF-α concentration induces leukocyte apoptosis, while sustained vascular inflammation elicits endothelial cell death (8). Apoptosis of cells of the respiratory tract and extra-pulmonary tissues is also directly activated by viral proteins (14, 31, 45, 46).

Unbalanced immune responses give rise to many of the pathologic hallmarks observed in respiratory virus-induced disease (5, 14). Apoptosis of epithelial and endothelial cells compromises alveolar-epithelial barrier leading to gas exchange impairment and fluid accumulation (30). Cytokine-induced uncontrolled epithelial cell replication, aberrant matrix remodeling, and fibrosis deposition perturb parenchyma architecture with further deterioration of respiratory function. Systemic manifestations are likewise dire components of the picture. Endothelial dysfunction significantly affects the physiological regulation of coagulation, shifting vascular equilibrium towards vasoconstriction, hypercoagulability, and immunothrombosis (9, 11). In addition, endotheliitis and consequent vascular leakage mediate translocation of inflammatory molecules as well as recruitment of activated neutrophils and cytotoxic T cells into peripheral tissue. Therefore, microvascular endothelial dysfunction, widespread immunothrombosis, and diffuse endothelial injury are significant contributing factors in development of tissue injury in extra-pulmonary sites (29, 30, 35, 43). Endothelial cell damage is likewise associated with unbalanced vasoactive molecule production leading to impaired blood pressure control. CD4+ and CD8+ T-cell lymphopenia is likely consequent to both virus-related direct effects (32) and hypercytokinemia-dependent induction of leukocyte death (4, 7, 14, 30). Finally, enhanced expression of IL-1 and/or other cytokines within the CNS could participate in T cell exhaustion and natural killer and B cell suppression observed in COVID-19 (32), leading to development of systemic immunosuppression (47).

This being said, it is important to mention that cytokines also contribute to the biological events required for a successful resolution of infection, including extracellular matrix remodeling and tissue repair (19). Therefore, their production should be modulated but not suppressed. In this view there is a clear therapeutic challenge aimed at reducing inflammation without canceling its defensive properties. Indeed, a balanced control of the deleterious virus/host interactions, is crucial to reduce damage and promote resolution; this strategy should complement direct anti-viral procedures.

## The Melanocortin System: A Potent Endogenous Modulatory Pathway

### Melanocortin Peptides and Their Receptors 

Melanocortins are a family of endogenous peptides produced by CNS and peripheral cells (25, 48, 49). These peptides derive from post-translational processing of the precursor proopiomelanocortin (POMC) and include the adrenocorticotropic hormone (ACTH) and the melanotropins, *α, β*, and *γ*-melanocyte-stimulating hormone (MSH).

Although *α*-MSH was initially described as a melanogenic hormone and activity of ACTH was confined to its steroidogenetic effect, it is now well established that melanocortins exert multiple actions on the host physiology. Indeed, over four decades, research has demonstrated that melanocortins have the remarkable ability to restore normal pathways when a certain stimulus perturbs the host homeostasis. Heterogeneous stimuli such as pathogens or their components, ischemia/reperfusion (I/R) injury, or irritants elicit production of melanocortin peptides that, in turn, act to restore equilibrium (25, 49, 50). Blockade of the natural peptides increases the expression of proinflammatory mediators in the blood, lungs, and liver of endotoxemic mice (51). These observations suggest that when endogenous production is not sufficient to face the challenge, supplementary administration could reach the target.

Melanocortin effects are exerted through recognition of five melanocortin receptors (MCRs) that are broadly distributed in peripheral cells and in brain regions. MCRs are G-protein-coupled receptors associated with adenylyl cyclase and mediate their effects primarily by activating the cAMP-PKA-CREB-dependent signaling pathway but also through activation of mitogen-activated-protein-kinase (MAPK), calcium-inositol triphosphate-PI3K, and JAK-STAT pathways (25, 52–54). With the exception of the MC2R, which is selectively activated by ACTH, the other MCR subtypes, MC1R, MC3R, MC4R, and MC5R, are recognized by all the natural melanocortins, although with different affinity (55). MC1R is widely expressed in body cells including fibroblasts, neutrophils, monocytes, B and T lymphocytes, dendritic cells, alveolar macrophages, glial cells, epithelial cells, and endothelial cells (25, 56–59). Binding affinity of endogenous agonists for this receptor is *α*-MSH=ACTH>β-MSH<γ-MSH (55). A large number of in vitro and in vivo studies demonstrate a primary role of MC1R in immunomodulation (25, 48, 49) as well as in regulation of endothelial cell function (57, 59). Of note, mice bearing a non-functional MC1R (Mc1r^e/e^) show exacerbated inflammatory responses relative to wild-type animals (60) and selective MC1R silencing in macrophages is associated with loss of *α*-MSH-mediated suppression of inflammation in vitro (61). Unlike the other MCRs, MC2R exclusively binds to ACTH that selectively induces glucocorticoid production in the adrenal cortex. MC3R is expressed in hypothalamus cells, macrophages, monocytes, dendritic cells, CD4+ T cells, and B lymphocytes and its binding ability to natural melanocortins is *γ*-MSH>ACTH=*α*-MSH=*β*-MSH (55). MC3R activation appears to be associated with broad modulation of inflammation in response to acute pro-inflammatory stimuli (62). MC4R [*α*-MSH=ACTH>*β*-MSH>*γ*-MSH (55),] is mainly expressed in the CNS and participates in immunodulation through activation of the cholinergic anti-inflammatory pathway (63). MC5R is widely expressed in both the CNS and in peripheral tissues, including the lung, kidney, lymph nodes, bone marrow, thymus, and immune competent cells such as B and T lymphocytes, mast cells, and antigen presenting cells (APC) (53, 64). Recent evidence suggests that MC5R is deeply involved in regulation of immune reactions and inflammatory responses (53, 65). Natural melanocortin order of potency in activating this receptor is: *α*-MSH>ACTH=*β*-MSH>>*γ*-MSH (55).

### Synthetic Ligands of MCRs

Development of synthetic derivatives of melanocortins may allow a better exploiting of the remarkable properties of the natural peptides due to their enhanced chemical stability, resistance to enzymatic degradation, and, for certain of them, more selective receptor recognition (66). A significant breakthrough in this approach was the synthesis of the *α*-MSH analog Nle4,DPhe7-*α*-MSH (NDP-*α*-MSH) marked by prolonged and increased biological activity compared to the endogenous peptide (67). NDP-*α*-MSH (afamelanotide) is clinically used to prevent phototoxicity in erythropoietic protoporphyria and could represent an excellent candidate for other melanocortin-based therapies. Additional *α*-MSH analogs include AP214 (modimelanotide) (68) and STY39 (69) that show higher affinity for MC1R/MC3R and for MC1R/MC5R, respectively. Of particular relevance with regard to clinical use, ACTH-related sequences form a solid basis for MCR-based effective therapies. Indeed, the extra-adrenal effects of ACTH are definitely recognized and these molecules have already been used to treat human inflammatory disorders (70–72).

Selective ligands for individual MCR subtypes are likewise being designed (66), including BMS-470539 (MC1R agonist) (73), [D-Trp8]-*γ*-MSH (MC3R) (74), RO27-3225 (MC4R) (75), PG-901 (MC5R) (54), and the N-terminally “capped” tetrapeptide 3,3,3-triphenylpropionyl-His-D-Phe-Arg-Trp-NH(2) (MC5R) (76).

### Protective Effects of Melanocortins in Systemic Inflammation and Secondary Organ Damage

Activation of the melanocortin system with natural or synthetic ligands exerts beneficial effects in acute, chronic, and systemic inflammatory disorders (25–27, 48, 49, 77). Moreover, different clinical studies have investigated efficacy of ACTH and NDP-*α*-MSH therapies in systemic inflammatory diseases, including ARDS, rheumatoid arthritis, multiple sclerosis (MS), lupus erythematosus (SLE), kidney diseases, and nephrotic syndromes (70–72).

**Table 1** reports the beneficial effects exerted by melanocortins in preclinical models of systemic inflammatory diseases, including sepsis, MODS, I/R injury, hemorrhage shock, and vasculitis.

**Table 1:** Protective effects exerted by melanocortin treatment in preclinical models of systemic inflammation and secondary organ damage.

MCR		Compound	Experimental model	Melanocortin treatment	Melanocortin effect	Ref.
**Systemic inflammation**						
	MC1R, MC3R, MC4R, MC5R	*α*-MSH	rabbit, LPS i.p.; blood collection and body temperature assessment at 0, 1, and 3 h	i.v. immediately before LPS administration	↓ fever	(50)
			mice septic shock (cecal ligation and puncture); survival at 12 and 24 h	i.p. at 0 and 3 h	improved survival rate	(77)
			mouse, LPS i.p.; blood collection at 1 and 6 h, liver and lung biopsy at 6 h	i.c.v. 15 min before LPS	↓ plasma TNF-α and nitrate	(51)
					↓ iNOS activity and iNOS mRNA in the lungs and liver	
					↓ lung myeloperoxidase activity	
			mouse peritonitis (monosodium urate crystals i.p.); peritoneal lavage at 4 and 6 h	s.c. 30 min before inflammatory challenge	↓ neutrophil migration	(78)
					↓ IL-1β and CXCL-1 in peritoneal lavage fluids	
		NDP-*α*-MSH	mouse peritonitis (zymosan i.p or monosodium urate crystals i.p.); peritoneal lavage at 4 and 6 h	s.c. 30 min before inflammatory challenge	↓ neutrophil migration	(79)
					↓ IL-1 β and CXCL-1 in peritoneal lavage fluids	
			mouse, LPS i.p. + zymosan i.p. after 6 d; lung biopsy and histology at 7 d, survival at 16 d	i.p. daily from 0 to 16 d	improved survival rate	(80)
					↓ inflammatory cells infiltrate into the lungs	
					↓ vascular congestion and interstitial edema in the lung tissue	
					↓ plasma TNF- α	
					↓ TNF- α and ↑ IL-10 mRNAs in the lungs	
		AP214	pig endotoxemia (LPS i.v.); cardiovascular monitoring from 20 to 180 min	i.v. at 0 h	↓ increase in pulmonary pressure and vascular resistance	(81)
		*α*-MSH, STY39	mouse, LPS i.p. + D-galactosamine; blood collection at time intervals, lung biopsy at 8 h	i.p. at 1, 2 or 3 h following LPS administration	↑ plasma TFPI	(82)
					↑ TFPI mRNA in the lungs	
	MC3R	*γ*2-MSH	mouse peritonitis (monosodium urate crystals i.p.); peritoneal lavage at 4 and 6 h	s.c. 30 min before inflammatory challenge	↓ neutrophil migration	(78)
					↓ IL-1 β in peritoneal lavage fluids	
**I/R injury**						
	MC1R, MC3R, MC4R, MC5R	NDP-*α*-MSH	mouse bilateral renal I/R (40 min ischemia + 4 8 or 24 h reperfusion); lung histology at 4 h after ischemia, lung biopsy at 0, 5, 4, and 8 h after ischemia	i.v. immediately before clamp removal and at 8 and 16 h post ischemia	↓ lung edema	(83)
					↓ leukocyte infiltration	
					↓ TNF- α and ICAM-1 mRNAs in the lungs	
					↓ lung myeloperoxidase activity	
					↓ IkB*α*, p38, and c-Jun phosphorylation in the lung tissue	
					↓ NF-kB and AP-1 DNA-binding activity in the lung tissue	
**Hemorrhagic shock**						
	MC1R, MC3R, MC4R, MC5R	NDP-α-MSH	rat, acute hemorrhagic shock; lung histology at 25 min and at 24 h, survival at 24 h	i.v. 5 min after termination of bleeding	improved survival rate	(84)
					improved cardiovascular parameters	
					↓ blood free radicals	
	MC4R	RO27-3225, PG-931	rat, acute hemorrhagic shock; lung histology at 25 min and 24 h, survival at 24 h	i.v. 5 min after termination of bleeding	improved survival rate	(84)
					improved cardiovascular parameters	
					↓ histological damage in the lungs	
					↓ blood free radicals	
		RO27-3225	rat, acute hemorrhagic shock; hemogasanalysis at 5, 15, and 60 min	i.v. 5 min after termination of bleeding	improved pH, pO_2_, pCO_2_, HCO_3_-, BE, SO_2_, and lactate	(85)
**Vasculitis**						
	MC1R, MC3R, MC4R, MC5R	*α*-MSH	mouse leukocytoclastic vasculitis (s.c LPS priming + i.p LPS after 24 h); ear histology at 24 h after LPS priming and at 2, 3, 5, 6 h after LPS	i.p. at 3 h after LPS priming	↓ vascular hemorrhage score	(86)
					↓ E-selectin and VCAM-1-positive vessels	
			mouse leukocytoclastic vasculitis (s.c. LPS priming + i.p. LPS after 24 h); ear histology at 2, 3, 5, 6 h after LPS	i.p. at 3 h after LPS priming	↓ vascular hemorrhage score	(87)
					↓ E-selectin and VCAM-1-positive vessels	
	MC1R	BMS-470539	mouse vasculitis (35 min mesenteric ischemia + 90 min reperfusion); intravital microscopy, mesentery tissue biopsy	i.v. before ischemia	↓ leukocyte adhesion and migration	(88)
					↓ CXCL1 and CCL2 mesenteric tissue expression	

BE, base excess; CXCL-1, chemokine (C-X-C motif) ligand 1; d, day; h, hour; HCO_3_-, bicarbonate; ICAM-1, intercellular adhesion molecule 1; IL-1β, interleukin-1β; IL-10, interleukin-10; IkBα, nuclear factor of kappa light polypeptide gene enhancer in B-cells inhibitor, alpha; iNOS, inducible nitric oxide synthase; i.p., intraperitoneal; I/R, ischemia/reperfusion; i.v., intravenous; LPS, lipopolysaccharide; min, minutes; pO_2_, partial pressure of oxygen; pCO_2_, partial pressure of carbon dioxide; s.c., subcutaneous; SO_2_, oxygen saturation; TNF-α, tumor necrosis factor α; TFPI, tissue factor protein inhibitor; VCAM-1, Vascular cell adhesion protein.

The marked protection in systemic inflammation was demonstrated by studies conducted in rabbits (50) and mice (51, 77–79). NDP-*α*-MSH administration in murine MODS not only increases survival rate but also reduces pulmonary leukocyte infiltration, vascular congestion, and interstitial edema (80). In a porcine model of systemic inflammatory response syndrome, treatment with the α-MSH analog AP214 prevented the LPS-induced increase in pulmonary pressure (81). *α*-MSH or STY39 administration in mice with endotoxemia is associated with increased concentration and mRNA pulmonary expression of TFPI (82).

In a model of renal I/R injury *α*-MSH administration prevented activation of NF-*k*B and AP-1 in the lungs and reduced the expression of stress response genes, intracellular adhesion molecule (ICAM)-1, and TNF-*α* (83). In addition, *α*-MSH significantly decreased leukocyte infiltration and lung edema.

Treatment with NDP-*α*-MSH or with the selective MC4 agonists RO27-3225 and PG-931 leads to restoration of cardiovascular and respiratory functions, improved survival, and reduced circulating free radicals in a rat model of severe hemorrhagic shock (84). Moreover, RO27-3225 prevents the hemorrhage-induced immunopathological changes in peripheral organs. Treatment with RO27-3225 normalizes hemogasanalysis parameters after hemorrhagic challenge (85).

Injury to vascular endothelium significantly contributes to convert a local inflammation into a systemic disease. In this perspective, the observation that *α*-MSH administration to endothelial cell challenged with pro-inflammatory stimuli is associated with reduced adhesion molecule expression (56, 87) and leukocyte adhesion (56, 87) has particular relevance. Further, melanocortin treatment reduced endothelial cell damage and barrier permeability in an *in vitro* model of blood-brain barrier inflammation (59). With regard to *in vivo* studies, Mc1r^e/e^ mice exposed to high-sodium diet or LPS challenge show increased susceptibility to inflammation-dependent vascular dysfunction (57). In leukocytoclastic vasculitis in mice, *α*-MSH significantly suppresses vascular damage and hemorrhage by inhibiting the early LPS-induced expression of E-selectin and VCAM-1 (86, 87). Activation of MC1R was associated with inhibition of cell adhesion/emigration and reduced tissue expression of CXCL1 and CCL2 in a murine model of vascular inflammation induced by I/R injury (88).

### Protective Effects of Melanocortins in Primary Inflammatory Disorders of the Lung 

Pulmonary cells express both MC1R (89, 90) and MC3R (89, 91). Expression of MC1R mRNA was documented in rat native lungs and a significant up-regulation of this receptor was observed in lungs subjected to *ex vivo* perfusion (92). As the procedure involves removal of non-resident cells from the lungs, this observation documents that MC1R is expressed by pulmonary tissue and is further induced during I/R injury.

In *in vitro* studies, *α*-MSH suppressed IL-1-induced PGE production by fetal human lung fibroblasts (93) and prevented LPS-induced activation of NF-*k*B in human pulmonary epithelial cells (94). The inhibitory effect is mediated by preservation of the IkB*α* protein (94). Similarly, *γ*-MSH treatment suppresses NF-*k*B signaling and exerts several protective effects in a human epithelial cell line challenged with different inflammatory stimuli (91).

The effectiveness of melanocortin treatment in control of primary lung inflammatory diseases is well documented (**Table 2**): this property could be very beneficial in the protection of organs that are a primary target of respiratory viruses.

**Table 2:** Protective effects exerted by melanocortin treatment in preclinical models of primary pulmonary inflammation.

MCR		Compound	Experimental model	Melanocortin treatment	Melanocortin effect	Ref.
**ALI/ARDS**						
	MC1R, MC3R, MC4R, MC5R	*α*-MSH	rat, LPS i.t.; BAL at 6 h	i.p. at 0, 2, and 4 h	↓ BAL WBCs	(77)
			rat ARDS (acute hemorrhagic shock + LPS i.t after 3 h); lung histology at 9 h post LPS	i.v. at 0, 3, and 6 h after LPS administration	↓ infiltration of inflammatory cells into alveoli	(95)
					↓ apoptosis of pulmonary endothelial cells	
			mouse, LPS i.n.; BAL at 4 h	i.p. 1 h before LPS administration	↓ BAL neutrophils	(89)
					↓ BAL TNF-α	
	MC1R	BMS-470539	mouse, LPS i.t.; BAL and lung biopsy at 18 h	s.c. 1 h before LPS administration	↓ lung W/D ratio	(96)
					↓ BAL leukocytes and PMNs	
					↓ BAL TNF-α	
					↓ lung myeloperoxidase activity	
	MC3R	[D-TRP8]-γ-MSH	mouse, LPS i.n.; BAL at 4 h	i.p. 1 h before LPS administration	↓ BAL neutrophils; ↓ BAL TNF-α	(89)
**ALI and fibroproliferative disorder**						
	MC1R, MC3R, MC4R, MC5R	NDP-α-MSH	rat, bleomycin i.t.; blood collection and lung biopsy at 8 and 24 h, lung W/D at 24 h	i.p. immediately before bleomycin instillation and at 12 h	↓ lung W/D ratio	(97)
					↓ expression of genes involved in stress response, fluid homeostasis, inflammation, fibrosis	
					↓ plasma nitrate	
		STY39	mouse, bleomycin i.t.; daily monitoring, lung index at 7 and 14 d, BAL at 7 d, lung biopsy at 9 d, lung histology at 14 d	i.p. from 1 to 14 d after bleomycin instillation	improved survival rate	(98)
					improved cyanosis, tachypnea	
					↓ BAL TNF-α, IL-6, MIP-2, and TGF-β1	
					↓ BAL macrophages, neutrophils, and lymphocytes	
					↓ type I and III procollagen pulmonary expression	
					improved MMP-1/TIMP-1 mRNA pulmonary ratio	
					↓ hydroxyproline and myofibroblast proliferation in the lung tissue	
**Airway allergic inflammation**						
	MC1R, MC3R, MC4R, MC5R	*α*-MSH	mouse OVA-induced allergic inflammation (i.p allergic sensitization at 1,14, and 21 d; aerosol challenge at 26 and 27 d); BAL and histology at 48 h	i.v. 30 min before each sensitization and each allergen challenge	↓ perivascular inflammation	(90)
					↓ peribronchial inflammatory cell infiltrate	
					↓ BAL eosinophils	
					↓ BAL IL-4, IL-13 and ↑ IL-10	
					↓ blood allergen specific IgE	
	MC3R	[D-TRP8]-*γ*-MSH	mouse OVA-induced allergic inflammation (i.p. allergic sensitization at 0 and 7 d, aerosol challenge at 14, 15, and 16 d); BAL at 24 h	i.p. 5 min before each allergen challenge	↓ BAL eosinophils and lymphocytes	(89)

BAL, bronchoalveolar lavage; d, days; h, hour; IL-4, interleukin-4; IL-6, interleukin 6; IL-10, interleukin-10; IL-13, interleukin-13; i.n., intranasal; i.p., intraperitoneal; i.t., intratracheal; i.v., intravenous; LPS, lipopolysaccharide; min, minutes; MIP-2, macrophage inflammatory protein 2; MMP-1, matrix metalloproteinase 1; OVA, ovoalbumin; PMNs, polymorphonuclear leukocytes; TGF-β1, transforming growth factor β1; TIMP-1, metallopeptidase inhibitor 1; TNF-α, tumor necrosis factor α; WBC, white blood cells; W/D, wet-to-dry.

In a preclinical model of ARDS based on intratracheal infusion of endotoxin, treatment with α-MSH was associated with reduced leukocyte count in BAL fluids (77). Similar salutary effects were observed in mice treated with *α*-MSH or a synthetic MC3R agonist after LPS administration (89). In an ARDS model based on induction of hemorrhagic shock followed by intratracheal LPS administration, *α*-MSH-treated rats showed reduced leukocyte infiltration and endothelial cell apoptosis (95). In LPS-induced ALI in mice, MC1R activation with the selective agonist BMS-470539 was associated with improved pulmonary edema, reduced inflammation and neutrophil infiltration (96).

Experiments based on intratracheal instillation of bleomycin are particularly interesting in that this model is widely used to reproduce human ALI and fibroproliferative disorders. A research based on bleomycin instillation in rats, showed that NDP-*α*-MSH administration markedly improves the clinical and molecular picture of ALI (97). In particular, NDP-*α*-MSH treatment was associated with a significant reduction of interstitial edema. At a molecular level, melanocortin treatment prevented bleomycin-induced increase in plasma NO concentration and modulated the expression of genes involved in stress response, inflammation, fluid homeostasis, and fibrosis development. Consistent with the observations that endogenous *α*-MSH participates in responses to host challenge, bleomycin caused a significant increase in circulating *α*-MSH relative to controls. The synthetic *α*-MSH analog STY39 (98), increased survival and improved the lung edema index in the bleomycin model. An interesting observation in this research is that STY39, in addition to inflammation, prevents bleomycin-induced pulmonary expression of pro-fibrotic factors and improves MMP-1/TIMP-1 mRNAs ratio.

A protective influence of melanocortins on altered cytokine production was likewise documented in a mouse model of allergic airway inflammation (90). Endogenous production of *α*-MSH in BAL after airway allergen challenge was observed. Treatment with *α*-MSH provided further benefit, including reduction in BAL eosinophils and a decrease in serum allergen-specific IgE, IL-4, and IL-5. Lower concentration of BAL eosinophils and lymphocytes was likewise reported by Getting and coworkers in allergic mice treated with [D-TRP8]-*γ*-MSH (89).

### Main Protective Pathways Influenced by Melanocortins

The anti-inflammatory effects of melanocortins are mainly exerted through PKA-mediated prevention of IκB*α* degradation and consequent inhibition of the NF-*k*B signaling in leukocytes (48, 99), endothelial cells (56, 59, 87), and other peripheral cells (48, 94). As NF-*k*B induces the expression of hundreds of genes relevant to inflammation including cytokines, cytokine receptors, chemokines, growth factors, and adhesion molecules, its modulation enables a wide-range regulation of inflammatory responses.

Besides the direct antiphlogistic effects, melanocortins have the peculiar feature to stimulate pro-resolving endogenous circuits (68, 70, 100–102). In fact, MCR activation is associated with induction of the expression of inhibitors of inflammation and resolutive factors, including IL-10 (103), IL-1 receptor-associated kinase (IRAK)-M (104), suppressor of cytokine signaling (Socs) 1 and 3 (101, 102), IkB*α* (102), cyclooxygenase (COX) 2 (102), Chemokine (C-C motif) ligand (CCL) 20 (101, 102), Interleukin 1 receptor antagonist (IL-1ra) (101, 102), Dual specificity phosphatase 1 (DUSP1) (105), and IL-1 receptor-like 2 (IL-1rl2) (105). MCR-induced resolving activities likewise include pro-efferocytic effects mediating clearance of apoptotic neutrophils (68).

Moreover, melanocortin peptides can activate immune regulatory mechanisms through induction of T cell differentiation toward a tolerogenic phenotype (106). In particular, Taylor and coworkers reported *α*-MSH-induced Treg cells are CD25+CD4+ and express CTLA4, CD44, CD62L, and latency-associated peptide (LAP) (65). Activation of regulatory activity in effector T cells and APCs was documented in a model of ocular inflammatory disease (64, 65, 106, 107). The modulatory effect on immunity could represent an additional mechanism through which melanocortin peptides promote resolution of inflammation (106).

There is increasing evidence that the diverse immunomodulating effects of melanocortins are carried out through specific MCR targeting. In fact, while resolution of inflammation is mainly achieved through activation of MC1R (25, 48, 49, 61) and MC3R (62, 78), immune regulation appears to be mediated by MC5R (64, 65, 108). As an instance, among the receptors expressed by macrophages, MC1R (61) and MC3R (68, 78) are involved in inflammatory response inhibition, whereas MC5R engagement promotes monocyte differentiation toward either myeloid suppressor cell or tolerogenic APCs with subsequent reduction of effector T cell activation (109).

A peculiar effect of melanocortin peptides consists of indirect influences exerted on peripheral cells through stimulation of MC3R/MC4R within the brain and activation of descending anti-inflammatory neural pathways (25, 48, 49, 110). Lipton and colleagues showed that *α*-MSH given centrally inhibits inflammation in the skin through activation of adrenergic pathways (110, 111). A further protective mechanism is based on MC4R signaling and subsequent stimulation of the cholinergic anti-inflammatory pathway (63) that inhibits inflammation in tissue macrophages and lymphoid organs.

Of note, due to its unique peptide sequence, ACTH elicits MC2R-mediated steroidogenesis in the adrenal gland. Therefore, in addition to the protective influences exerted through activation of the other MCRs, ACTH induces glucocorticoid production (70). This additional effect can provide a remarkable clinical advantage as glucocorticoids and melanocortins exert distinct effects as immune modulators (112). Consequently, ACTH can provide combined beneficial effects based on two different pathways. In fact, glucocorticoids can be exploited to induce a general suppression of immune cell activity, inhibit pro-inflammatory mediator release, promote leukocyte apoptosis, and prevent cell recruitment into damaged tissue. On the other hand, melanocortin peptides, including ACTH, are able to modulate rather than dampen the release of inflammatory mediators, elicit production of resolutive factors, induce leukocyte differentiation towards protective phenotype, and exert a considerable anti-microbial activity (113, 114). The distinctive features of glucocorticoids- and melanocortins-based therapies are particularly manifest in the treatment of ocular inflammatory disorders in which *α*-MSH administration not only suppresses eye inflammation, but also induces immune tolerance and promotes retinal cell survival (115).

Finally, another potentially beneficial effect exerted by melanocortins is protection against apoptotic cell death. This action was observed in pulmonary vascular endothelial cells in a model of ARDS (95), in macrophages exposed to serum starvation *in vitro* (116), and in a rat model of prolonged myocardial I/R (117).

## Discussion: Exploiting Melanocortin Pathways to Counteract Respiratory Virus-Induced Detrimental Events

As stated above, respiratory viruses can alter inflammatory reactions built up by the host to combat the infection. In this scenario, a potentially beneficial cytokine production can turn into a dysregulated harmful event that eventually contributes to development of the severe clinical manifestations observed in CoVs and IAVs-induced diseases. Therefore, modulation of the host aberrant inflammatory responses appears as crucial as controlling viral replication to prevent disease progression.

Melanocortins could reinforce endogenous signals providing protection *versus* respiratory virus-induced detrimental events. In particular, disruption of the dire and self-amplificating vicious-cycle triggered by mediator release/leukocyte recruitment can significantly reduce pulmonary tissue damage. Through modulation of NF-*k*B signaling, melanocortin peptides do not influence a single inflammatory mediator but they rather exert control on the whole inflammatory cascade. This effect could be of key importance during respiratory virus infection. Evidence obtained in SARS-CoV-1-infected mice clearly demonstrated that chemical blockade of NF-*k*B improves lung pathology, reduces inflammation, and increases survival (40). Moreover, melanocortin-induced expression of negative regulators of inflammation as well as the activation of immune regulation mechanisms can counteract pro-inflammatory signals and trigger repair mechanisms crucial to restore homeostasis.

The possibility to induce glucocorticoid production is another peculiar feature inherent in therapeutic activation of melanocortin pathways. Availability of ligands with different affinities to MCRs provides the unique opportunity to either take advantage of or avoid adrenal stimulation, depending on the specific patient clinical situation. In fact, similar to steroid treatment, overproduction of endogenous cortisol can be associated with adverse side effects including increased susceptibility to infections, hypertension, diabetes mellitus, electrolyte disturbances, gastric ulceration, and impaired wound healing (118). On the other hand, based on the most recent guidelines on the use corticosteroid-based therapy in COVID-19, severe and critical patients could significantly benefit from glucocorticoid production (24). Therefore, the use of ACTH requires a preventive assessment to determine the balance between benefits and harms of enhancing corticosteroids (118). Further, it is important to consider that ACTH-induced steroidogenesis produces glucocorticoid plasma concentrations that could be insufficient when high dose steroid therapy is needed (115). Conversely, *α*-MSH and its analogs can be safely administered when adrenal stimulation should be avoided.

In addition to immunomodulation, melanocortin treatment could ameliorate pulmonary edema and fibrosis. Of note, experiments in rodent liver fibrosis demonstrated that melanocortin therapy not only can prevent the biological events leading to fibrosis development but it can also reverse established fibrosis and exert a collagenolytic effect (119). Melanocortin-mediated inhibition of pulmonary endothelial cell apoptosis could likewise contribute to preserve alveolar-epithelial barrier integrity. Moreover, pharmacological activation of MCRs can exert a protective effect against endothelial inflammation and aberrant activation of coagulation cascades that substantially contribute to disease progression in respiratory virus infections.

Finally, targeting melanocortin system allows exploiting the beneficial effects exerted by the cholinergic anti-inflammatory pathway on systemic inflammation and deranged coagulation (120). In addition, the central neurogenic influences of melanocortins and their effects on descending anti-inflammatory neural pathways could be of paramount importance in view of a potential immunosuppression elicited by virus-induced inflammatory mediator release within the CNS (121).

Therefore, the central and peripheral protective actions exerted following MCR activation could collectively allow dampening the harmful events that trigger the cytokine storm and endothelial dysfunction while sustaining the beneficial signals required to elicit resolution and repair mechanisms.

## Concluding Remarks

Enhancing endogenous protective responses to viral infection while inhibiting harmful signals is a key approach to prevent disease progression to a critical life-threatening state, particularly in the absence of specific antiviral drugs. We deem that translational use of melanocortin molecules could be an exploitable strategy in this setting. The key to the unique beneficial modulatory effects induced by melanocortins resides in their capacity to reduce the aberrant responses to infection without impairing the host defense mechanisms. Despite several overlapping influences, this represents a major distinction relative to the other potent, endogenous anti-inflammatory system formed by glucocorticoids. Capacity to take advantage of a protective endogenous system, deeply explored over the years, could help to face present and future emergencies marked by severe pathogen/host interactions.

## Author Contributions

All authors listed have made a substantial, direct, and intellectual contribution to the work and approved it for publication.

## Conflict of Interest

The authors declare that the research was conducted in the absence of any commercial or financial relationships that could be construed as a potential conflict of interest.
